# Influence of an Organic Salt‐Based Stabilizing Additive on Charge Carrier Dynamics in Triple Cation Perovskite Solar Cells

**DOI:** 10.1002/advs.202304502

**Published:** 2023-10-09

**Authors:** Patrick Dörflinger, Yong Ding, Valentin Schmid, Melina Armer, Roland C. Turnell‐Ritson, Bin Ding, Paul J. Dyson, Mohammad Khaja Nazeeruddin, Vladimir Dyakonov

**Affiliations:** ^1^ Experimental Physics 6 Julius Maximilian University of Würzburg 97074 Würzburg Germany; ^2^ Institute of Chemical Sciences and Engineering École Polytechnique Fedérale de Lausanne (EPFL) Lausanne 1015 Switzerland

**Keywords:** microwave conductivity, mobile ions, mobility, perovskite solar cell, stability

## Abstract

Besides further improvement in the power conversion efficiency (PCE) of perovskite solar cells (PSC), their long‐term stability must also be ensured. Additives such as organic cations with halide counter anions are considered promising candidates to address this challenge, conferring both higher performance and increased stability to perovskite‐based devices. Here, a stabilizing additive (*N*,*N*‐dimethylmethyleneiminium chloride, [Dmmim]Cl) is identified, and its effect on charge carrier mobility and lifetime under thermal stress in triple cation perovskite (Cs_0.05_MA_0.05_FA_0.90_PbI_3_) thin films is investigated. To explore the fundamental mechanisms limiting charge carrier mobility, temperature‐dependent microwave conductivity measurements are performed. Different mobility behaviors across two temperature regions are revealed, following the power law T^m^, indicating two different dominant scattering mechanisms. The low‐temperature region is assigned to charge carrier scattering with polar optical phonons, while a strong decrease in mobility at high temperatures is due to dynamic disorder. The results obtained rationalize the improved stability of the [Dmmim]Cl‐doped films and devices compared to the undoped reference samples, by limiting temperature‐activated mobile ions and retarding degradation of the perovskite film.

## Graphical abstract

A stabilizing additive for triple‐cation perovskite solar cells is identified that increases both performance and stability. These observations are supported by temperature‐dependent investigations of charge carrier mobility using microwave conductivity and open‐circuit voltage decay.

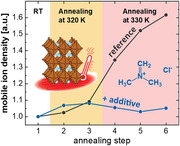

## 1 Introduction

In the past decade, perovskite solar cells (PSC) evolved into one of the most promising photovoltaic materials, with steadily rising power conversion efficiencies (PCE) now over 26% in the leading examples.^[^ ^1^ ^]^ The outstanding and promising opto‐electronic properties of perovskites, such as a tunable bandgap, high absorption coefficient, and long charge carrier lifetime have enabled this rapid success.^[^ ^2^, ^3^, ^4^, ^5^, ^6^ ^]^ Nonetheless, organometal halide perovskites suffer from insufficient long‐term stability, which is an essential requirement for the commercialization of PSCs.^[^ ^7^ ^]^

In the past, several mechanisms responsible for the deterioration of perovskite films have been reported, such as light‐induced decomposition or thermal degradation, or phase segregation in mixed halide perovskites.^[^ ^5^, ^8^ ^]^ Different approaches to improve the stability have been pursued, using various organic and inorganic components mixed into the precursor solution or as an additional layer.^[^ ^9^ ^]^ For example, substitution of the A‐site cation can result in improvements in the charge carrier mobility, and the incorporation of rubidium and cesium ions yields a reduction in trap densities.^[^ ^10^, ^11^, ^12^ ^]^ Different Lewis bases are able to passivate uncoordinated Pb^2+^ ions, and several organic additives forming low‐dimensional perovskites show improvements toward moisture‐ and phase‐stability with passivating effects.^[^ ^13^, ^14^, ^15^ ^]^ Using ammonium salts, such as methyl ammonium chloride (MACl) as an additive can improve crystallization, surface morphology, and charge carrier lifetime.^[^ ^16^ ^]^ Especially the use of additives with chlorine anions as counterparts are of interest, as they show improvements such as increased grain size, suppressing undesired side phases or increasing crystallinity by retarding the growth rate.^[^ ^17^, ^18^, ^19^ ^]^ Starting from the early use of MACl, the trend tends to be more functionalized additives with additional mechanisms. Hence, ionic liquids and other organic‐based salts are promising additives to improve long‐term stability further.^[^ ^20^ ^]^ For example, incorporation of 1‐(4‐ethenylbenzyl)‐3‐(tridecafluorooctyl)‐imidazolium iodide (ETI) into MAPbI_3_ gave a greater resistance toward thermal degradation and increased hydrophobicity.^[^ ^21^ ^]^ Furthermore, addition of 1‐butyl‐3‐methylimidazolium tetrafluoroborate (BMIMBF_4_) to triple cation perovskites showed not only an improvement in stability, but also in device performance.^[^ ^22^ ^]^

In this report, we describe the effect of *N*,*N*‐dimethylmethyleneiminium chloride ([Dmmim]Cl) as a dopant on the optoelectronic properties of the perovskite and photovoltaic performance of devices, focusing on time‐resolved microwave conductivity (TRMC) measurements to obtain insights into the charge carrier dynamics. We find that the dopant [Dmmim]Cl increases the intrinsic stability of PSCs under operating conditions and reduces the temperature‐activated mobile ions while maintaining high charge carrier mobility and lifetime.

## 2 Results and Discussion

### 2.1 Impact of [Dmmim]Cl on Charge Carrier Dynamics

**Figure 1:**
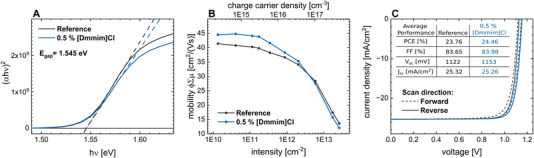
Band gap and mobility characterization of the reference and [Dmmim]Cl‐doped perovskite and device performance. a) Tauc‐plots of the reference and [Dmmim]Cl‐doped perovskite used to determine the band gap of both thin films. The reference and the doped perovskites exhibit a band gap of 1.545 eV. b) Quantum yield φ times the sum of electron and hole mobility Σµ as a function of laser fluence and injected charge carrier density. The quantum yield φ decreases with increasing laser intensity due to the fast recombination of charge carriers. Mobility is extracted at the plateau obtained at low laser fluences, showing that the [Dmmim]Cl‐doped perovskite exhibits a slight increase in charge carrier mobility compared to the reference perovskite. c) *J–V* characteristics of the solar cells with and without the dopant, in forward and reverse scan direction. The average solar cell parameters are collected in a table in the inset. The concentration of the [Dmmim]Cl in the precursor is 0.5 mol %.

As a reference, a triple cation halide perovskite (cesium methylammonium formamidinium lead triiodide, Cs_0.05_MA_0.05_FA_0.90_PbI_3_) was prepared, and for the doped samples 0.5 mol % [Dmmim]Cl was added directly into the perovskite precursor solution before spin coating (see Supporting Information S1). The perovskite composition provides an optimized bandgap of 1.545 eV due to the high amount of formamidinium and suppresses the formation of the non‐perovskite hexagonal δ‐FAPbI_3_ phase.^[^ ^23^, ^24^ ^]^ Furthermore, the use of pure iodine avoids halide segregation.^[^ ^25^, ^26^ ^]^ As the incorporation of ionic salt into the perovskite lattice can influence the band gap, we performed absorption measurements and extracted the bandgap from the Tauc‐plot (**Figure** **1**a; for calculation, see Supporting Information S2). Both perovskite films with and without [Dmmim]Cl dopant exhibit the same bandgap of E_gap_ = 1.545 eV, which is in line with literature values for comparable perovskite compositions.^[^ ^23^, ^27^ ^]^ Supporting steady‐state photoluminescence measurements show no influence of the dopant on the PL (see Supporting Information S2).

TRMC measurements were performed to gain deeper insight into the intrinsic charge carrier properties. TRMC is a contactless measurement technique, allowing us to probe the photo‐excited charge carriers in a thin film without the influence of additional contacts. A microwave resonator is used to generate a standing electromagnetic wave, whose electric field component is maximum at the point where the thin film is located. This resonant approach allows further enhancement of the sensitivity of the measurement. The charge carriers in the sample are generated by a laser pulse, which changes the conductance of the thin film. The interaction of free charge carriers with the microwaves leads to a change in the microwave field distribution inside the resonator, which results in a microwave reflection and can be used as a measurable which mimics the charge carrier dynamics. From the measured conductance (G), the sum of the electron and hole mobility (Σµ) can be calculated as shown in Equation (1):

(1) $$\phi \Sigma \mu \;=\frac{\Delta G_{\mathrm{Max}}}{e\beta IF_{A}}\;$$

where *φ* is the yield of free carrier generation, e is the elementary charge, β the ratio of resonator width to height, *F*_A_ the fraction of absorbed photons, I the laser intensity and Δ*G*_Max_ the maximum value of the change in conductance. Due to charge carrier recombination, the change in conductance Δ*G* decreases with time. This transient behavior is directly proportional to the charge carrier density Δ*n* decay, and gives us information about the charge carrier lifetime. A detailed description of the TRMC setup used in this work and subsequent analysis can be found in Supporting Information S4.

As shown in Figure 1b, the product of quantum yield ϕ and the sum of electron and hole mobility Σμ of the reference and doped perovskite thin film is plotted for different excitation intensities. The apparent decrease of ϕΣμ from low excitation intensities at 10^10^ cm^−2^ toward high excitation intensities at 10^13^ cm^−2^ is related to the decrease of the quantum yield and has its origin in the specific response time of the cavity‐based measurement technique.^[^ ^28^ ^]^ The higher excitation power leads to a higher charge carrier density. Hence, the charges recombine faster, i.e., with a higher recombination rate.^[^ ^29^ ^]^ When a significant number of charge carriers recombine at times shorter than the response time of the setup, the remaining charges available in the perovskite are fewer than calculated in Equation (1) (*n*  =  *I* × *F*_A_), resulting in a decreasing ϕΣμ. To obtain the correct mobility value, the quantum yield must be close to unity, which is a good approximation at low excitation intensities. Therefore, the mobility reaches a maximum at low fluences when a quantum yield of one is reached (Figure 1b). The mobility of the reference perovskite was determined to be 41.4 cm^2^ V^−1^ s^−1^, whereas the [Dmmim]Cl doped perovskite showed a slight increase in mobility up to 45.4 cm^2^ V^−1^ s^−1^, which is comparable to literature values of similar triple cation compositions.^[^ ^11^ ^]^ The corresponding transient decay is shown in Supporting Information S5. A slight increase in mobility could be an effect of the chlorine anion of the [Dmmim]Cl, which is known to increase the grain size of the perovskite film.^[^ ^18^ ^]^ This is supported by scanning electron microscope measurements, revealing an increased grain size for the doped perovskite. (see Supporting Information S3). The decays taken at similar laser intensities show a similar trend for the doped and undoped (reference) perovskite films and thus no significant change in the recombination dynamics can be revealed. Figure 1c shows the impact of the [Dmmim]Cl additive on the current density–voltage (*J–V*) curves characteristics of fully processed solar cells measured under one sun illumination (see Supporting Information S6). The *J–V* curves correspond to the champion cells, and the device parameter in the inset display the average values of a statistic of 28 solar cell devices (see Supporting Information S6 and Tables S2 and S3). The open‐circuit voltage *V*_OC_ improves slightly, whereas the short‐circuit current *J*_SC_ and the fill‐factor (FF) remains unaffected, resulting in power conversion efficiencies of (23.76 ± 0.12) % for the reference device and (24.46 ± 0.11) % for the [Dmmim]Cl‐doped device. Additional maximum power point tracking shows a stable power output for both devices (see Supporting Information S6). Thus, the [Dmmim]Cl additive in the perovskite absorber does not lead to a shift in the optical band gap but to a slight increase in mobility, as well as comparable device performance. Moreover, the external quantum efficiency spectra for the reference and [Dmmim]Cl‐doped perovskite solar cells show no substantial differences, confirming the identical *J*_SC_ values (see Supporting Information S7). High FF values of ≈84% are observed in both cases, and hence the addition of [Dmmim]Cl into the perovskite precursor solution apparently has no detrimental effect.

**Figure 2:**
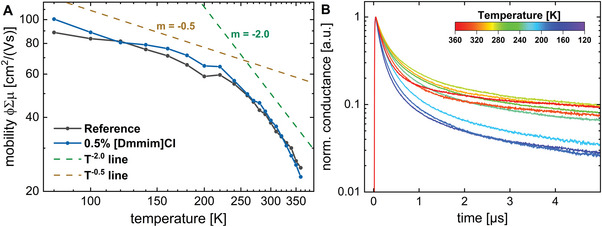
Temperature‐dependent charge carrier mobility and transients. a) Charge carrier mobility at different temperatures is deduced from TRMC measurements. At low temperatures the slope of *m* = −0.5 indicates the scattering of charge carriers on optical phonons, whereas the slope of *m* = −2.0 at higher temperatures can be assigned to dynamic disorder. Since both curves coincide, the [Dmmim]Cl dopant does not seem to effect mobility. b) Corresponding transients to the mobility measurement in (a) for the reference perovskite, excited with a laser intensity of 3.6 × 10^11^ photons per cm^2^ which corresponds to a charge carrier density of 6.5 × 10^15^ cm^−3^.

To gain further insight into charge carrier dynamics, the temperature‐dependent behavior of the mobility for perovskite films was studied with and without [Dmmim]Cl dopant. The obtained power‐law of the temperature‐dependent mobility *μ* *∝* *T* ^*m*^ is indicative of the dominant scattering process that limits the maximum achievable mobility.^[^ ^30^ ^]^ In **Figure** **2**a the quantum yield times sum of mobility, ϕΣμ, is plotted against the temperature in the range of T = 80 to 360 K. The charge carrier mobility behaves very similarly in both perovskite films and is strongly temperature‐dependent increasing from 20 to 100 cm^2^ V^−1^ s^−1^ toward lower temperatures. Interestingly, the temperature‐dependence follows a power‐law of *μ* *∝* *T* ^*m*^, and reveals two distinguishable slopes, denoted by the dashed lines in Figure 2a. Between 80 and 220 K, the mobility dependence displays a slope of *m* ≈ −0.5, followed by a transition toward a steeper slope of *m* ≈ −2.0 above 240 K, and continuing this trend to 360 K.

Previous studies showed that the scattering processes in hybrid and inorganic lead halide perovskites are ruled by the interaction of charge carriers with longitudinal optical phonons.^[^ ^31^, ^32^ ^]^ These Fröhlich interactions, forming large polarons, suggest a power‐law of *m* ≈ −0.5, matching our observations well in the temperature range from 80 to 220 K.^[^ ^33^, ^34^ ^]^ This behavior has also been demonstrated experimentally on pure FAPbI_3_ perovskite with a slope of *m* ≈ −0.53.^[^ ^33^ ^]^ For pure inorganic CsPbI_3_ a power‐law of *m* ≈ −0.77 was found, which agrees moderately well with the predicted value of −0.5.^[^ ^32^ ^]^ In contrast, early studies on MAPbI_3_ consistently revealed steeper slopes in the range between *m* =  −1.5 and −1.8 in the tetragonal phase, supporting the view that scattering of electrons by acoustic phonons (*m* ≈ −1.5) is the predominant mobility limiting mechanism.^[^ ^35^, ^36^, ^37^ ^]^ Such discrepancies from the theoretical value were explained not only by intrinsic electron‐phonon coupling but also by extrinsic effects, such as temperature‐activated barriers to the charge transport.^[^ ^38^ ^]^ Thus the high FA content of 90% of the A‐site cation seems to be responsible for the low‐temperature slope of *m* ≈ −0.5 (Figure 2a), whereas the influence of the MA content seems negligible, as this would result in a slope *m* ≥ −1.5. However, for temperatures above 240 K, the mobility starts to decrease faster, resulting in a slope of *m* ≈ −2.0. This can be explained by the appearance of dynamic disorder in the crystal lattice.^[^ ^39^, ^40^ ^]^ Fluctuations in the Pb‐I bond length due to the high temperature lead to large variations in the orbital overlap, resulting in new dominant scattering mechanisms, and a greater limitation of the maximum achievable mobility. This is reflected by a steeper slope of mobility at higher temperatures. The theoretically obtained slope of *m* ≈ −2.1 fits well with the data in Figure 2a.^[^ ^39^ ^]^ As dynamic disorder is caused by the metal‐halide lattice, it should be independent of the A‐site cation.^[^ ^38^ ^]^ Hence, [Dmmim]Cl should not influence the scattering mechanism or the obtained slope in the high‐temperature range under operational conditions. As confirmed in Figure 2a, no influence of [Dmmim]Cl on the charge carrier mobility is observed. In order to investigate the influence of dynamic disorder on charge carrier recombination, the TRMC transients were also analyzed at different temperatures. In Figure 2b, similarities are observed compared to the mobility in Figure 2a. The transient decay is governed by the recombination (*R*) of charge carriers, which strongly depends on the charge carrier density (*n*), as seen from Equation (2).

(2) $$R\;=\;-\;\frac{dn}{dt}=\;nk_{1}+n^{2}k_{2}+n^{3}k_{3}$$

Here, *t* is the time, *k*_1_ is the monomolecular recombination rate constant, *k*_2_ is the bimolecular recombination rate constant, and *k*_3_ is the Auger recombination rate constant. At low charge carrier densities, monomolecular recombination dominates, whereas for larger charge carrier densities, higher order recombination mechanisms prevail. As the decay in Figure 2b was measured at an injected charge carrier density equal to one sun illumination, it is mainly influenced by mono‐ and bimolecular recombination, while Auger recombination may be neglected.^[^ ^29^ ^]^ From 360 to 280 K the transient decay is prolonged, which can primarily be seen in the initial decay below 1 µs. However, between 240 and 280 K, a sudden transition in the transient decay occurs, leading to a gradual faster decay with lowering of the temperature to 140 K. The change in the temperature‐dependent recombination dynamics coincides with the observed change in the power‐law of mobility. Therefore, we suggest that the temperature induced dynamic disorder affects not only the scattering mechanisms, but possibly also the recombination dynamics. Studies on the recombination dynamics of MAPbI_3_ confirm the observed behavior in Figure 2b, showing an initial decrease and then a subsequent increase in the bimolecular recombination rate upon lowering the temperature.^[^ ^36^ ^]^ Furthermore, transient photoluminescence of a similar perovskite composition exhibits comparable changes in the transient behavior.^[^ ^41^ ^]^ Finally, below 140 K the decay is slowed down (see Supporting Information S8). This may be due to a phase transition, which alters the recombination dynamics, but shows no discontinuity of the mobility as is the case for MAPbI_3_.^[^ ^37^ ^]^ It has previously been shown that for pure FA or mixed cation perovskites with major FA content, a phase transition at around 150 K occurs, which is in agreement with our observations.^[^ ^41^, ^42^ ^]^ Comparing the differences between the reference and the [Dmmim]Cl doped film (see Supporting Information S8), similar temperature‐dependent behavior on the recombination and mobility is observed. Nonetheless, the initial charge carrier decay of the doped perovskite is slightly slowed, which indicates a passivation effect.

### 2.2 Influence of [Dmmim]Cl on Stability Under Thermal Stress

**Figure 3:**
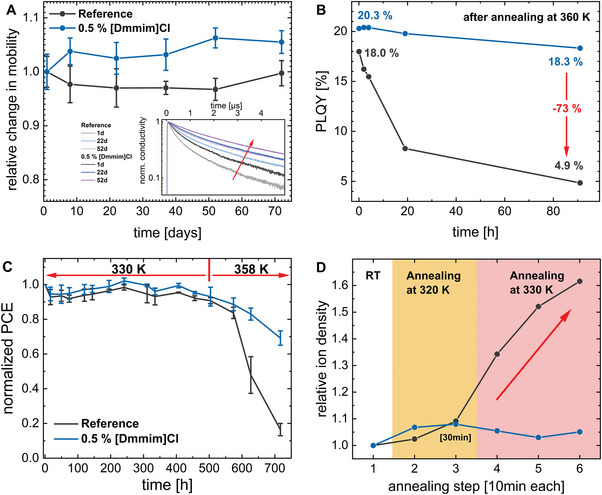
Investigations of long‐term and thermal stability, including mobility, PLQY and OCVD measurements and PCE tracking. Same color coding for all Figures. a) Change in mobility over 72 days, revealing a slight increase in mobility for the [Dmmim]Cl doped perovskite compared to the reference. The inset shows the corresponding transients, which exhibit reduced recombination over time for both samples. b) PLQY for both samples after thermal annealing at 360 K. The PLQY of the reference thin film decreases quickly, whereas the doped perovskite resists the thermal stress. c) Normalized PCE of the reference and doped device stored at 60 then 85 °C. For higher temperatures, [Dmmim]Cl reduces degradation due to thermal stress. d) Mobile ion density in the perovskite device was measured with OCVD. The graph displays the mobile ion density at room temperature after different annealing steps with increasing temperature. The reference exhibits a gradual increase of mobile ion density at room temperature after the solar cell has been annealed at 330 K.

To further explore the influence of [Dmmim]Cl doping on stability, the effect of thermal annealing on the optoelectronic properties of thin films and devices was investigated. The stability of the reference and [Dmmim]Cl‐doped perovskite films was monitored at room temperature, allowing possible degradation over time or due to illumination during the thermal stress measurements to be determined.^[^ ^8^ ^]^ Therefore, perovskite thin films were stored for 72 days in a nitrogen‐filled glovebox, and the mobility was measured at regular intervals. In **Figure** **3**a, the relative change in the charge carrier mobility of the reference film shows an unchanged mobility, whereas the doped perovskite interestingly exhibits a slightly increasing mobility. In general, the mobility of a perovskite thin film is expected to be a robust parameter under inert conditions, whereas temperature cycling, illumination‐driven decomposition, or phase segregation can negatively affect the charge carrier mobility of a perovskite. However, the underlying mechanism remains unclear and requires further investigation. Consequently, the investigated triple cation perovskite and the [Dmmim]Cl doped thin film show no signs of degradation due to the storage and/or the measurement itself.

Next, photoluminescence quantum yield (PLQY) measurements were conducted to obtain a qualitative description of the recombination dynamics (see Supporting Information S9). The PLQY consists of an interplay of the three recombination rates as follows:

(3) $$\mathrm{PLQY}\;=\frac{n^{2}k_{2}}{nk_{1}+n^{2}k_{2}+n^{3}k_{3}}\;=\frac{dn/dt_{\mathrm{rad}}}{dn/dt_{\mathrm{tot}}}\;$$

Here, d*n*/d*t*_rad_ denote the radiative recombining charge carriers and d*n*/d*t*_tot_ the total recombination. The PLQY is determined from the charge carrier density *n* in the perovskite film and the three recombination rate constants, monomolecular recombination *k_1_*, bimolecular recombination *k_2_* and the Auger recombination *k_3_*. Using a continuous‐wave laser with a power comparable to one sun illumination, *k_3_* has only a minor influence and can be neglected.^[^ ^29^ ^]^ As the illumination intensity is kept constant, *n* does not vary. Thus, any change in the PLQY is attributed to changes in *k_1_* and *k_2_*. For the reference perovskite, a PLQY of 18.0% was achieved, whereas the [Dmmim]Cl‐doped perovskite showed a PLQY of 20.3%. Subsequently, the thin films were heated to 360 K for four days, with the PLQY measured periodically at room temperature to investigate the effect of heating. As shown in Figure 3b, the reference sample shows a decrease in the PLQY of 73%. In contrast, the [Dmmim]Cl‐doped perovskite loses only 10% of its initial PLQY value. As *k_2_* is an intrinsic material property, it should remain constant during the measurements.^[^ ^43^ ^]^ In addition, Davies et al. demonstrated that bimolecular recombination is an inverse absorption process.^[^ ^44^, ^45^ ^]^ Since the absorption of the thin films remain constant after annealing (Supporting Information S9, Table S1), while maintaining their black color, implies that the absorption property of the films is not affected by thermal annealing and thus the *k_2_* stays constant. Consequently, only an increase in *k_1_* can explain the decreasing PLQY, according to Equation (2). The increase in *k_1_* may be caused by additional non‐radiative trap‐assisted recombination, due to annealing of the thin film. To connect the increase of non‐radiative recombination with device performance, the quasi‐Fermi level splitting (QFLS) can be calculated from the change in PLQY according to Equation (3), as the QFLS represents the maximum achievable V_OC_ in a solar cell:^[^ ^46^ ^]^

(4) $$\Delta \mathrm{QFLS}\;=k_{B}T\times \ln\left({\mathrm{PLQY}}_{1}\right)-k_{B}T\times \ln\left({\mathrm{PLQY}}_{2}\right)$$

For the [Dmmim]Cl‐doped perovskite film, the change in PLQY after annealing results in a decrease in QFLS of 3 meV, whereas the loss in QFLS for the reference is 34 meV. As a consequence, this effect should be seen as a negative influence on device performance and can be linked to defect‐related losses in the device and therefore, to a lower open‐circuit voltage *V*_OC_. The monomolecular charge carrier recombination mostly originates from trap‐assisted recombination, showing that the [Dmmim]Cl dopant effectively prevents an increase of trap‐assisted recombination after annealing.^[^ ^47^ ^]^ A possible mechanism for the rise of non‐radiative recombination is described below.

Having demonstrated that the triple cation perovskite film exhibits higher stability both at room temperature and under solar cell operational temperatures upon doping with a small amount of [Dmmim]Cl, a fully processed solar cell was prepared to determine whether the increased stability translates to the device as a whole. Therefore, two devices (one doped with [Dmmim]Cl and one undoped) were prepared, and annealed on a hotplate at T = 333 K for 500 h with periodic measurement of their PCE. The procedure resulted in a slight reduction of the PCE in both devices. However, both solar cells sustained over 90% of their initial PCE value (Figure 3c). As no severe deterioration occurred, the temperature was increased to 358 K for another 200 h, resulting in a more substantial decrease of the PCE in both solar cells. The reference solar cell loses more than 80% of its efficiency, whereas doping of the perovskite with [Dmmim]Cl limits the degradation of the solar cell to a loss of only 30% of its initial PCE value. The difference in PCE between the reference and [Dmmim]Cl‐doped solar cells after thermal annealing at 358 K may be mainly attributed to the stabilizing effect of the [Dmmim]Cl, while, for example, degradation of the transport layers during the annealing can worsen the efficiency of both solar cells.^[^ ^48^, ^49^ ^]^

The presence of mobile ions has been connected to an accelerated degradation of solar cell devices.^[^ ^8^ ^]^ Therefore, open‐circuit voltage decay (OCVD) measurements were performed to gain further insight into the possible mechanism responsible for solar cell degradation induced by thermal stress. For the OCVD measurement, a voltage bias of 1.0 V was applied to the solar cell until it reached a steady state. After switching off the bias, the voltage decay at the solar cell is tracked over time, which allows to calculate the mobile ion concentration *N*_ion_ in the device (see Supporting Information S10 for further details).^[^ ^50^, ^51^ ^]^ For the reference solar cell an ion concentration of 1.4 × 10^18^ cm^−3^ was obtained whereas the solar cell doped with [Dmmim]Cl showed a slight reduction of the ion concentration to 1.2 × 10^18^ cm^−3^. Both values are consistent with literature values for the same device architecture.^[^ ^52^ ^]^

Similar to the measurement on perovskite thin films, the solar cells were heated to a specific temperature for a given time. After cooling to room temperature, the devices were remeasured. The behavior of the mobile ion concentration is plotted in Figure 3d. We deliberately started with a low temperature of 320 K and short annealing times of 10 min, as cells often display changes more rapidly than thin films.^[^ ^8^ ^]^ For both devices, an increase in the ion concentration of < 10% was observed. By slightly increasing the annealing temperature to 330 K, the ion concentration of the reference device began to increase after each temperature cycle. Finally, a 60% increase of the ion density N_ion_ after the third annealing step was achieved. An accumulation of ions at the perovskite/charge transport interface can capture free charge carriers and thereby accelerate the non‐radiative recombination. The large number of mobile ions after annealing may be attributed mainly to the degradation of the perovskite itself, and thus an increasing ion density in the bulk.^[^ ^8^ ^]^ To investigate the origin of the degradation, X‐ray diffraction (XRD) measurements were conducted, to identify structural changes in the perovskite (see Supporting Information S11). The peak corresponding to PbI_2_ in the reference perovskite film increases progressively with the heating time to a greater extent than observed in the [Dmmim]Cl doped film. The growth in the PbI_2_ peak indicates more significant decomposition of the perovskite.^[^ ^53^, ^54^, ^55^ ^]^ Unfortunately, at temperatures above 330 K the series resistance of the devices strongly increases, which prevents OCVD measurements at higher temperatures (see Supporting Information S10). Nonetheless, the significant increase of mobile ion concentration as evidence of degradation in the reference perovskite strongly suggests a general reduction in the long‐term stability under thermal stress. In contrast, the doped device showed no further increase of the ion density and remained unaffected by the heating cycle, confirming that temperature‐activated mobile ions are inhibited by doping the perovskite with [Dmmim]Cl. Presumably, the additive increases the activation energy of the mobile ion migration through lattice distortions due to the variation of the dopant molecule size.^[^ ^56^ ^]^ Earlier we saw that, the transient TRMC decay showed only minor differences at room and higher temperatures (working conditions, see Supporting Information S10) for the reference and [Dmmim]Cl‐doped perovskite. In contrast, the solar cell showed higher efficiency due to an increased open circuit voltage, which would change the recombination dynamics, which is not seen in the perovskite thin film. The difference here could be in the sample geometry since, in a solar cell, the accumulated mobile ions can affect the recombination dynamics, which is not seen in a pure perovskite thin film.

## 3 Conclusion

[Dmmim]Cl was used as an additive in the preparation of perovskite thin films and shown to improve the stability of solar cells under operating conditions. The dopant has only a minor impact on charge carrier dynamics and optical properties. Temperature‐dependent measurements of the charge carrier mobility reveal two temperature regions with different power‐law dependences, indicating different scattering mechanisms. The low‐temperature regime may be assigned to charge carrier scattering with polar optical phonons, whereas at high temperatures the strong decrease in mobility is attributed to dynamic disorder. We found that [Dmmim]Cl does not influence the observed power‐law. Annealing steps revealed the extraordinary stability of the [Dmimm]Cl‐doped perovskite toward higher temperatures up to 360 K, which is essential for the long‐term stability of solar cells, whereas the undoped reference undergoes significant degradation. With the incorporation of [Dmimm]Cl, the stability of the perovskite toward thermal stress was increased without detrimental effects on the performance of the solar cell, providing a step toward commercialization.

## 4 Experimental Section

### Materials

Lead(ii) iodide (PbI_2_; 99.99%, TCI), titanium diisopropoxide bis(acetylacetonate (75%, Sigma–Aldrich), methylammonium chloride (MACl; 99.99%, Greatcell solar), formamidinium iodide (FAI; 99.99%, Greatcell solar), methylammonium iodide (MAI; 99.99%, Greatcell solar), phenethylamine iodide (PEAI; 99.99%, Greatcell solar), *N*,*N*‐dimethylformamide (DMF; 99.8%, Sigma–Aldrich), dimethyl sulfoxide (DMSO; 99.9%, Sigma–Aldrich), 2‐propanol (99.5%, Sigma–Aldrich), chlorobenzene (99.8%, Sigma–Aldrich), Spiro‐OMeTAD (Borun Tech.), 4‐tert‐butylpyridine (tBP; Sigma–Aldrich), bis(trifluoromethane)sulfonimide lithium salt (Li‐TFSI; 99.95%, Sigma–Aldrich), tris(2‐(1H‐pyrazol‐1‐yl)−4‐tert‐butylpyridine)cobalt(III) tri[bis(trifluoromethane)sulfonimide] (FK209 Co(iii) TFSI salt Sigma–Aldrich), and acetonitrile (ACN; 99.8%, Sigma–Aldrich).

### Fabrication of Perovskite Solar Cells and Thin Films

Devices with an architecture of FTO glass/compact TiO_2_ layer (c‐TiO_2_)/mesoporous TiO_2_ layer (meso‐TiO_2_)/Cs_0.05_MA_0.05_FA_0.9_PbI_3_ (PVK)/PEAI/ spiro‐OMeTAD (HTM)/Au structure were fabricated. The patterned FTO substrate (Asahi FTO glass, 12–13 Ω cm^−2^) was sequentially cleaned with detergent (5% Hellmanex in water), deionized water, acetone, and isopropanol in the ultrasonic bath for 30 min, respectively. Then, a c‐TiO_2_ blocking layer was deposited on the FTO glass by spray‐coating the precursor solution consisting of a titanium diisopropoxide bis(acetylacetonate) solution in isopropanol (5% v/v), followed by sintering at 450 °C for 20 min (c‐TiO_2_). After cooling, single‐crystal TiO_2_ paste were spin‐coated on the compact TiO_2_ layer to prepare m‐TiO_2_ layer, and then sintered in air at 500 °C for 30 min, thereby obtaining the meso‐TiO_2_ layer.^[^ ^57^ ^]^ After sintering, the c‐TiO_2_/m‐TiO_2_ were ready to use and transferred into a glovebox for preparing the perovskite layer. The perovskite precursor solution (1.4 m) was prepared by adding 645.4 mg of PbI_2_, 216.7 mg of formamidium iodide (FAI), 11.1 mg of methylamonium iodide (MAI), and 11.8 mg of CsCl into 200 µL of *N*,*N*'‐dimethylsulfoxide (DMSO) and 800 µL of dimethyformamide (DMF) mixture. The solution was then stirred for 2 h at 60 °C. For the [Dmmim]Cl doped perovskite solution, 0.5 mol % of [Dmmim]Cl was added into the perovskite precursor solution. After UV‐ozone treatment of the substrates for 15 min, the perovskite precursor solution was spin‐coated onto the surface of the FTO/c‐TiO_2_/meso‐TiO_2_ substrate at 1000 rpm for 10 s, accelerated to 5000 rpm for 5 s and maintained at this speed for 20 s. This process was carried out in an N_2_‐filled glove box. Then, the substrate was placed in a home‐made rapid vacuum drying equipment, as previously reported.^[^ ^58^ ^]^ After pumping for 20 s, a brown, transparent perovskite film with a mirror‐like surface was obtained. The fresh perovskite layer was annealed at 100 °C for 1 h and then at 150 °C for 10 min. Afterward, 60 µL of PEAI solution (5 mg mL^−1^ in isopropanol) was spin‐coated on the perovskite film at 5000 rpm for 30 s. A hole transport layer was deposited on the perovskite film by depositing a doped spiro‐OMeTAD solution at 3000 rpm for 30 s. The doped spiro‐OMeTAD solution was prepared by dissolving 105 mg of spiro‐OMeTAD and 41 µL of 4‐tert‐butylpyridine in 1343 µL of chlorobenzene with additional 25 µL of bis(trifluoromethane)sulfonimide lithium salt solution (517 mg mL^−1^ in acetonitrile) and 19 µL of cobalt‐complex solution (376 mg mL^−1^ in acetonitrile). Finally, a ≈70 nm‐thick gold layer was evaporated on the spiro‐OMeTAD layer as the back electrode. The perovskite thin films for UV–vis, PLQY, and TRMC measurements were fabricated on sapphire substrates using the same preparation route as described for the solar cells.

### Characterizations

Absorption measurements were performed on thin films using a PerkinElmer Lambda 950 instrument with an integrating sphere. The mobility was measured with a time‐resolved microwave conductivity setup. For optical excitation a laser (Ekspla PL2210) with 532 nm was used. Neutral density filters were used to adjust the excitation power, measured with a power meter (Newport 843‐R‐USB power meter and 818‐UV/DB sensor). For the microwave cavity, a rectangular waveguide was used with a grating on both sides. As this setup measures in reflectance (one antenna for microwave in‐ and output), a circulator was needed to separate the reflected from the incident microwaves and guide them to the detection unit. The latter consisted of a tunnel diode detector (Aeroflex ACTP‐1504) converting the microwave power into a DC current, which was converted into a voltage by a high‐speed FEMTO amplifier (DHPCA‐100). For the temperature‐dependent measurements, a continuous flow cryostat was used to cool the whole resonator with the sample. After optical excitation of the sample, the change of photoconductance was monitored with a digital oscilloscope. From the maximum change of conductance between dark and illuminated sample the mobility could be determined. A more detailed description can be found in the Supporting Information.

*J–V* measurements were performed on a Keithley model 2400 digital source meter controlled by Test point software under a xenon lamp (450 W Xenon, AAA class). The light intensity was calibrated with a NREL‐certified KG5‐filtered Si reference diode. The active area of small cells was masked with a metal aperture of 0.09 cm^2^. An anti‐reflection coating layer was employed for measuring devices. All *J–V* curves of small devices were measured using a reverse scan (from 1.20 to 0 V) and a forward scan (from 0 to 1.20 V) under a constant scan speed of 10 mV s^−1^.

For the PLQY measurements, an integrating sphere in an Edinburgh Instruments FLS 980 was used. The sample was excited with a 635 nm CW laser diode with 98.7 mW cm^−2^. The spot size was 0.046 cm^2^.

OCVD measurements were performed by biasing the solar cell with 1.0 V in forward direction for a duration of 100 s and rapidly switching off the bias using a homemade switch and an Agilent 81150A function generator. Subsequently, the open circuit voltage was monitored over a 1 TΩ input impedance amplifier (Femto Messtechnik GmbH) and with an Agilent Infinium 90254A digital storage oscilloscope. Dark *J–V* measurements were recorded with a 6430 Sub‐Femtoamp Remote Sourcemeter (Keithley). A more detailed description can be found in the Supporting Information.

## Conflict of Interest

The authors declare no conflict of interest.

## Supporting information

Supporting Information

## Data Availability

The data that support the findings of this study are available from the corresponding author upon reasonable request.
