# Supplementary material for: Influence of an Organic Salt‐Based Stabilizing Additive on Charge Carrier Dynamics in Triple Cation Perovskite Solar Cells
Source: Adv Sci (Weinh). 2023 Oct 9;10(34):2304502. doi: 10.1002/advs.202304502 (PMC10700180; doi:10.1002/advs.202304502)
Supplement: Supplementary file 1 — Supporting Information [file ADVS-10-2304502-s001.pdf]

ADVANCED SCIENCE
Open Access

Supporting Information

for *Adv. Sci.*, DOI 10.1002/advs.202304502

Influence of an Organic Salt-Based Stabilizing Additive on Charge Carrier Dynamics in Triple Cation Perovskite Solar Cells

*Patrick Dörflinger, Yong Ding, Valentin Schmid, Melina Armer, Roland C. Turnell-Ritson, Bin Ding, Paul J. Dyson, Mohammad Khaja Nazeeruddin and Vladimir Dyakonov**

Supporting Information

Influence of an organic salt-based stabilizing additive on charge carrier dynamics in triple cation perovskite solar cells

*Patrick Dörflinger, Yong Ding, Valentin Schmid, Melina Armer, Roland C. Turnell-Ritson, Bin Ding, Paul J. Dyson, Mohammad Khaja Nazeeruddin and Vladimir Dyakonov**

P. Dörflinger, V. Schmid, M. Armer, V. Dyakonov
Experimental Physics 6, Julius Maximilian University of Würzburg, 97074 Würzburg, Germany
E-mail: vladimir.dyakonov@uni-wuerzburg.de

Y. Ding, R. C. Turnell-Ritson, B. Ding, P. J. Dyson, M. K. Nazeeruddin
Institute of Chemical Sciences and Engineering, École Polytechnique Fedérale de Lausanne (EPFL), 1015 Lausanne, Switzerland

Keywords: stability, perovskite solar cell, microwave conductivity, mobile ions, mobility

S1 Film and device preparation

Materials: The materials used include: lead(ii) iodide (PbI_2; 99.99%, TCI), titanium(iv) chloride ($TiCl_4$; 99%, Sigma-Aldrich), hydrochloric acid (HCl; 37 wt% in H_2O, Sigma-Aldrich), methylammonium chloride (MACl; 99.99%, Greatcell solar), formamidinium iodide (FAI; 99.99%, Greatcell solar), methylammonium iodide (MAI; 99.99%, Greatcell solar), phenethylamine iodide (PEAI; 99.99%, Greatcell solar), N,N-dimethylformamide (DMF; 99.8%, Sigma-Aldrich), dimethyl sulfoxide (DMSO; 99.9%, Sigma-Aldrich), 2-propanol (99.5%, Sigma-Aldrich), chlorobenzene (99.8%, Sigma-Aldrich), Spiro-OMeTAD (Borun Tech.), 4-tert-butylpyridine (tBP; Sigma-Aldrich), bis(trifluoromethane)sulfonimide lithium salt (Li-TFSI; 99.95%, Sigma-Aldrich), tris(2-(1H-pyrazol-1-yl)-4-tert-butylpyridine)cobalt(III) tri[bis(trifluoromethane)sulfonimide] (FK209 Co(iii) TFSI salt Sigma-Aldrich), and acetonitrile (ACN; 99.8%, Sigma-Aldrich).

Fabrication of perovskite solar cells and thin films: Devices with an architecture of FTO glass/compact TiO_2 layer (c-TiO_2) /mesoporous TiO_2 layer (meso-TiO_2)/$Cs_{0.05}MA_{0.05}FA_{0.9}PbI_3$ (PVK)/PEAI/ spiro-OMeTAD (HTM)/Au structure were fabricated. The patterned FTO

substrate (Asahi FTO glass, 12-13 Ω cm^{-2}) was sequentially cleaned with detergent (5% Hellmanex in water), deionized water, acetone, and isopropanol in the ultrasonic bath for 30 min, respectively. Then, a c-TiO_2 blocking layer was deposited on the FTO glass by spray-coating the precursor solution consisting of a titanium diisopropoxide bis(acetylacetonate) solution in isopropanol (5% v/v), followed by sintering at 450 °C for 20 min (c-TiO_2). After cooling, single-crystal TiO_2 paste were spin-coated on the compact TiO_2 layer to prepare m-TiO_2 layer, and then sintered in air at 500 °C for 30 min, thereby obtaining the meso-TiO_2 layer.[1] After sintering, the c-TiO_2/m-TiO_2 were ready to use and transferred into a glovebox for preparing the perovskite layer. The perovskite precursor solution (1.4 M) was prepared by adding 645.4 mg of PbI_2, 216.7 mg of formamidium iodide (FAI), 11.1 mg of methylamonium iodide (MAI), and 11.8 mg of CsCl into 200 μL of N, N'-dimethylsulfoxide (DMSO) and 800 μL of dimethyformamide (DMF) mixture. The solution was then stirred for 2 h at 60 °C. For the [Dmmim]Cl doped perovskite solution, 0.5 mol % of [Dmmim]Cl was added into the perovskite precursor solution. After UV-ozone treatment of the substrates for 15 min, the perovskite precursor solution was spin-coated onto the surface of the FTO/c-TiO_2/meso-TiO_2 substrate at 1000 rpm for 10 s, accelerated to 5000 rpm for 5s and maintained at this speed for 20 s. This process was carried out in an N_2 filled glove box. Then, the substrate was placed in a home-made rapid vacuum drying equipment, as previously reported.[2] After pumping for 20 s, a brown, transparent perovskite film with a mirror-like surface was obtained. The fresh perovskite layer was annealed at 100 °C for 1 h and then at 150 °C for 10 min. Afterwards, 60 μL of PEAI solution (5 mg/mL in isopropanol) was spin-coated on the perovskite film at 5000 rpm for 30 s. A hole transport layer was deposited on the perovskite film by depositing a doped spiro-OMeTAD solution at 3000 rpm for 30 s. The doped spiro-OMeTAD solution was prepared by dissolving 105 mg of spiro-OMeTAD and 41 μL of 4-tert-butylpyridine in 1343 μL of chlorobenzene with additional 25 μL of bis(trifluoromethane)sulfonimide lithium salt solution (517 mg/mL in acetonitrile) and 19 μL of cobalt-complex solution (376 mg/mL in acetonitrile). Finally, a ~70 nm-thick gold layer was evaporated on the spiro-OMeTAD layer as the back electrode. The perovskite thin films for UV/Vis, PLQY and TRMC measurements were fabricated on sapphire substrates using the same preparation route as described for the solar cells.

S2 Absorption and photoluminescence measurements

The absorption measurements for the Tauc-plots were performed on thin films using a PerkinElmer Lambda 950 instrument with an integrating sphere. For each sample the transmission T and reflection R was measured. To calculate the absorption coefficient α (Figure S1a) for a thin film, we used Lambert-Beers law $\alpha = -\ln(T)/d$, where d is the film thickness.

The photoluminescence measurements were performed on an Edinburgh Instruments FLS 980. The sample was excited with a 635nm CW laser diode with 98.7 mW/cm^2. The spot size was 0.046 cm^2. The corresponding photoluminescence spectra is seen in Figure S1b.

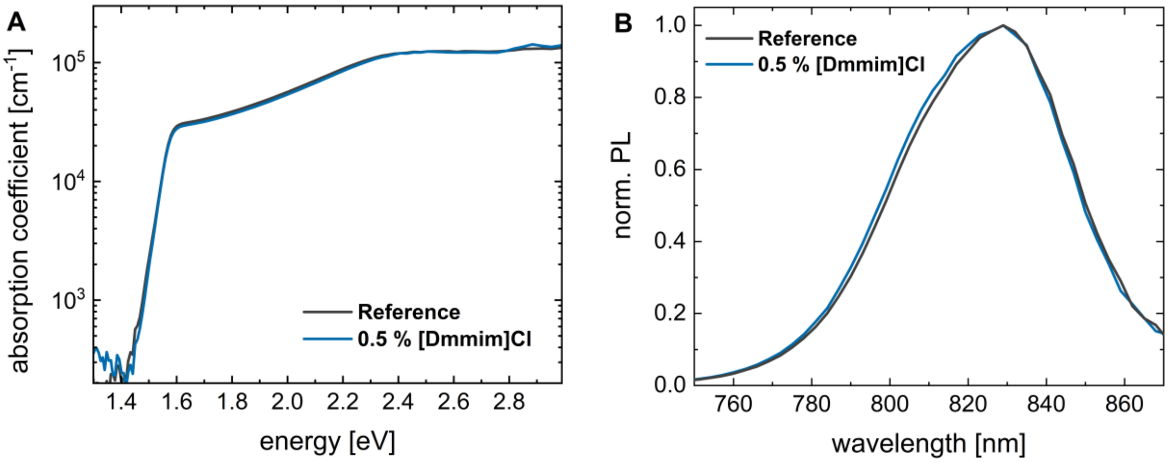

Figure S1: Optical properties of the [Dmmim]Cl doped and undoped perovskite thin film. (a) Absorption coefficient and (b) photoluminescence of the 0.5 mol % [Dmmim]Cl doped and undoped perovskite thin film does not indicate any changes in optical properties.

S3 Morphological characterization with scanning electron microscope (SEM)

The morphology of perovskite films was investigated by using scanning electron microscopy (SEM, FEI Sirion-200) with a voltage of 5 kV and a current of 0.1 nA. In Figure S2a and b the reference and [Dmmim]Cl doped perovskite film is shown, no significant alteration of the morphology can be seen. However, the mean grain size is slightly increased for the [Dmmim]Cl-doped perovskite as seen in the inset of Figure S2c, especially the smaller grain sizes are reduced. This appearance is consistent with the increased mobility seen by TRMC measurements. In addition, in Figure S2b a possible spot where grain coalescence may occur is marked.

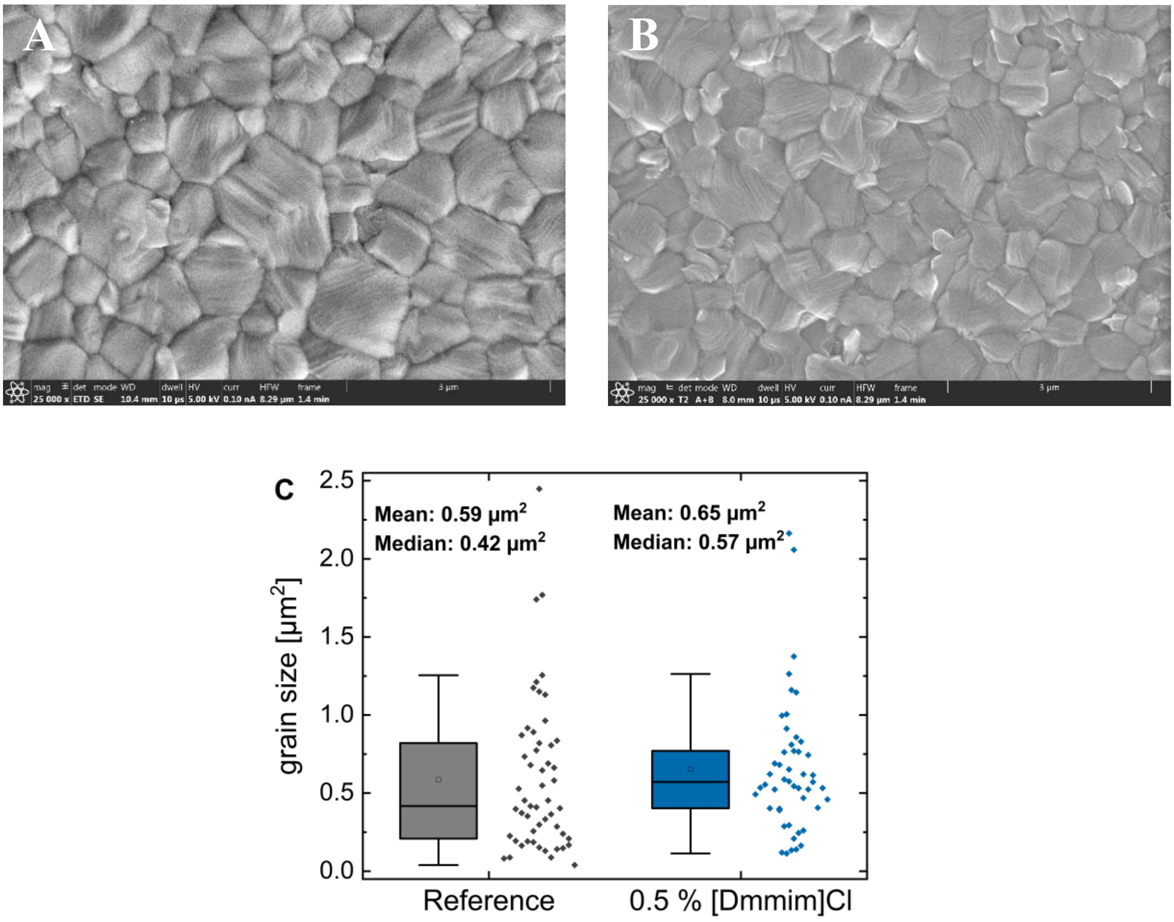

Figure S2: Morphology of the [Dmmim]Cl doped and undoped perovskite thin film and the grain size distribution. SEM pictures of the **(a)** reference and the **(b)** [Dmmim]Cl-doped perovskite. (c) The grain size distribution shows a slight increase for the [Dmmim]Cl-doped perowskite.

S4 Time-resolved microwave conductivity (TRMC) setup and measurements

Time-Resolved Microwave Conductivity (TRMC) is a resonant and contactless measurement technique to determine the mobility of photo-generated charge carriers as well as obtain information about their decay. As TRMC can be measured in different configurations, we will shortly describe the used setup and analysis. In Figure S3, a scheme of the three basic modules of the setup is shown. A pulsed laser (Ekspla PL2210), the microwave cavity (home-build) and the microwave source (Wiltron 69137A) and detection unit (GaGe high-speed digitizer oscilloscope).

For the microwave cavity we use a rectangular waveguide with a grating on both sides. For the microwave in- and output a hook is placed inside the resonator. The cavity operates in the transverse electric mode TE_{102} and forms a standing wave at approximately 9.6 GHz. In its field

maximum at ¾ of the resonator length L, the thin film is placed, as can be seen in Figure S3a. The use of a cavity enhances the sensitivity of the measurement, as the microwaves form a standing wave and increases the electric field strength in its node. As this setup measures in reflectance (one antenna for in- and output), a circulator is needed to separate the reflected from the incident microwaves and guides them to the detection unit. The latter consists of a tunnel diode detector (Aeroflex ACTP-1504) converting the microwave power into a DC current, which is converted into a voltage by a high-speed FEMTO amplifier (DHPCA-100). For the temperature-dependent measurements, a continuous flow cryostat was used to cool the whole resonator with the sample.

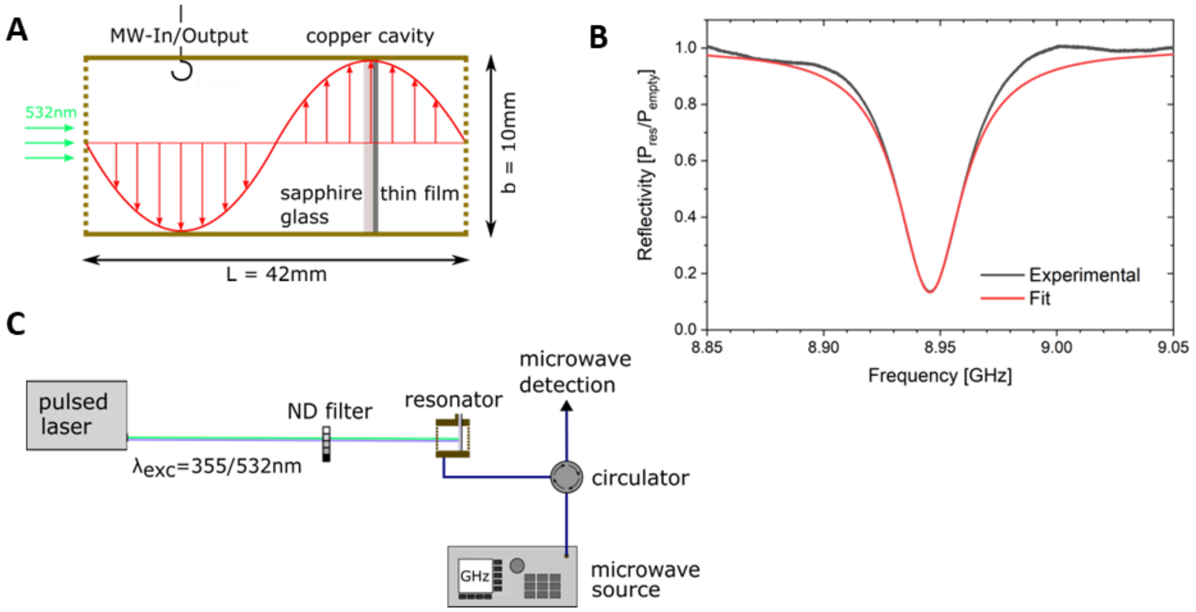

Figure S3: TRMC setup and resonance measurements. (a) TRMC cavity with the standing wave pattern in red. **(b)** Resonance curve of the cavity and the corresponding Lorentzian fit. **(c)** TRMC setup with the cavity, laser and microwave generation and detection unit.

First, a dark frequency sweep of the reflected microwaves is needed, shown in Figure S3b. The sweep reveals the resonance frequency of approx. 8.945 GHz of the cavity with the perovskite film on a substrate. As the substrate and the perovskite film exhibit a $\varepsilon_r > 1$, they will increase the optical pathway and thus decrease the resonance frequency. Subsequent, the experimentally obtained resonance curve is fitted by a Lorentzian function to obtain the cavity parameters, like the resonance frequency f_0, the full width at half of the minimum ΔW and the resonance depth R_0. From these parameters the sensitivity factor K is calculated, which is needed to determine the mobility.

$$K = \frac{2Q_L\left(-1+1/\sqrt{R_0}\right)}{\pi f_0 \varepsilon_0 \varepsilon_r d\beta} \qquad \text{(SE1)}$$

ε_0 is the dielectric constant, ε_r the relative permittivity of the material filling the cavity, d is the length of the cavity, β is the ratio of resonator width a to height b of the cavity. The quality factor Q_L can be determined by the resonance parameter according to Equation SE2. The response time τ is another important quantity determine the temporal solution. Since the quality factor increases the sensitivity factor K as well as the response time, the interplay between the sensitivity and the response time needs to be considered.

$$Q_L = \frac{f_0}{\Delta W} \qquad \text{(SE2)} \qquad\qquad \tau = \frac{Q_L}{\pi f_0} \qquad \text{(SE3)}$$

To measure the mobility, the microwave input is set to the resonance frequency and the appropriate power is adjusted. The charge carriers, generated with the second harmonic of a Nd:YAG laser (532 nm), lead to an increase of the conductance G in the perovskite film. The normalized change of reflected microwave power ΔP/P from the resonator as measurand is connected according to Equation SE6 with the change in conductance ΔG between dark and illuminated sample and a sensitivity factor K, obtained by additional resonance measurements as shown before.

Due to charge carrier recombination, the change in conductance ΔG is decreasing and the signal decays, as shown in Figure S4a for different excitation intensities. This transient behavior is directly proportional to the charge carrier density Δn in the film and provides information about the charge carrier recombination. For high excitation intensities, the transient decay accelerates which is indicative for higher order recombination. To finally extract the sum of the electron and hole mobility Σμ (SE5), the maximum change of the conductance ΔG_{Max}, the absorption F_A of the perovskite film, the illumination intensity I and β as the ratio of the resonator dimensions is needed. e is the elementary charge.

$$\frac{\Delta P}{P} = K \cdot \Delta G \quad \text{(SE4)} \qquad\qquad \phi\Sigma\mu = \frac{\Delta G_{Max}}{(e\beta I_0 F_A)} \qquad \text{(SE5)}$$

The mobility values obtained from Equation SE5 are plotted against the excitation intensity in Figure S4c. It seems that the mobility decreases towards higher excitation intensities. This can be explained by the fact, that for the mobility calculation the maximum change in photo-conductance ΔG_{Max} is used. Due to the resonance characteristic of the measurement method, a significant amount of charge carriers can recombine within the response time. This leads to a discrepancy between the measured ΔG_{Max} and the excited charge carrier density ($n = I*F_A$) used for the mobility calculation. This effect is visualized in Figure S4b, when the linear proportionality between ΔG_{Max} and the excitation intensity I given by Equation SE5 is invalid. As the recombination accelerates with the charge carrier density in the thin film, this effect

dominates at high laser fluences. For this reason, the sum of the mobility of the target material is extracted when the mobility values saturates.

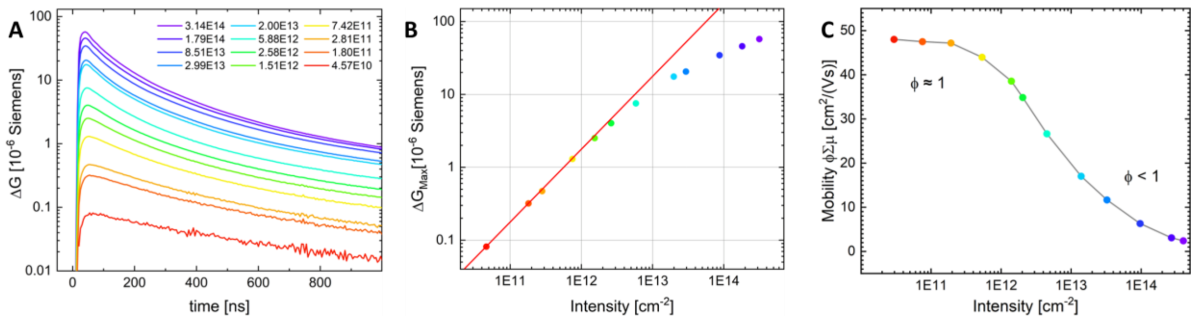

Figure S4: Exemplary TRMC transients, extracted conductance and mobility at different intensities. (a) TRMC transients for different excitation intensities. The transition from first-order to second-order decay with increasing intensities can be seen. **(b)** Change in conductance due to the photo-generated free charge carriers for different excitation intensities. The red solid line with a slope of 1 indicates the linear proportionallity of intensity and change in conductance, which is valid for low excitation intensities. **(c)** Extracted mobility versus the excitation intensitiy. The artificial decrease of the mobility for higher excitation intensities is mainly due to recombination of charge carriers within the response time of the setup, influencing the mobility calculation.

The formation of excitons can also artificially reduce the ΔG_{Max} as only free charges are contributing. This should be considered, especially for materials with high exciton binding energies. For this reason, the quantity ϕ as a quantum yield is introduced, which is close to unity for low excitation fluences. Nonetheless, the decay on higher charge carrier densities gives insights in the change in recombination dynamics.

S5 Room temperature TRMC transients

The photo-excited charge carriers in the perovskite film immediately starts to recombine through different recombination mechanisms, strongly depending on the charge carrier density n. For low charge carrier densities, monomolecular charge carrier recombination prevails. As the exciton binding energy is low in triple cation perovskites, we assume that monomolecular recombination originated from trap-assisted recombination.[3; 4] For higher charge carrier densities, bimolecular band-to-band recombination starts to dominate, which is proportional to the square of charge carrier density. By further increasing the charge carrier density, Auger recombination becomes dominant. A many-body process involving three particles.

$$R = -\frac{\mathrm{d}n}{\mathrm{d}t} = nk_1 + n^2k_2 + n^3k_3 \quad \text{(SE6)}$$

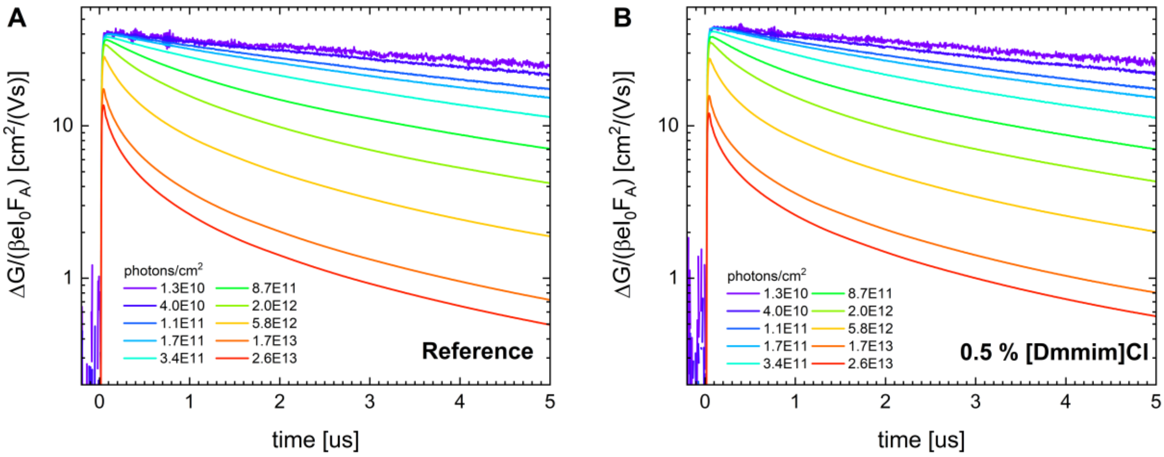

Figure S5: TRMC transients of the reference and [Dmmim]Cl-doped thin film for different excitation intensities. Transient decay of the reference (left) and [Dmmim]Cl-doped (right) perovskite. For small charge carrier densities n in the perovskite, achieved by a low excitation intensity, the decay of both samples shows a monomolecular dominated recombination. At higher excitation intensities, a faster decay component appears, resulting in a faster decay. Comparing the transient behavior of both samples, the doping of [Dmmim]Cl shows no strong impact on the recombination dynamics.

Supporting Table S1: Conversion of the laser fluence in photons/cm^{-2} in injected charge carrier density cm^{-3}.

**Laser fluence in photons/cm^2**	**Injected charge carrier density cm^{-3}**
1.3E10	2.3E14
4.0E10	7.0E14
1.1E11	1.9E15
1.7E11	3.0E15
3.4E11	6.0E15
8.7E11	1.5E16
2.0E12	3.5E16
5.8E12	1.0E17
1.7E13	3.0E17
2.6E13	4.6E17

S6 Current density-voltage measurements and maximum power point (MPP) tracking

The current density-voltage (J-V) measurements were performed on a Keithley model 2400 digital source meter controlled by Test point software under a xenon lamp (450 W Xenon, AAA class). The light intensity was calibrated with a NREL-certified KG5-filtered Si reference diode. The active area of small cells was masked with a metal aperture of 0.09 cm^2. An anti-reflection coating layer was employed for measuring devices. All J-V curves of small devices were measured using a reverse scan (from 1.20 V to 0 V) and a forward scan (from 0 V to 1.20 V) under a constant scan speed of 10 mV/s. All device parameters are displayed in Figure S6, and the single values are listed in Table S2 and S3.

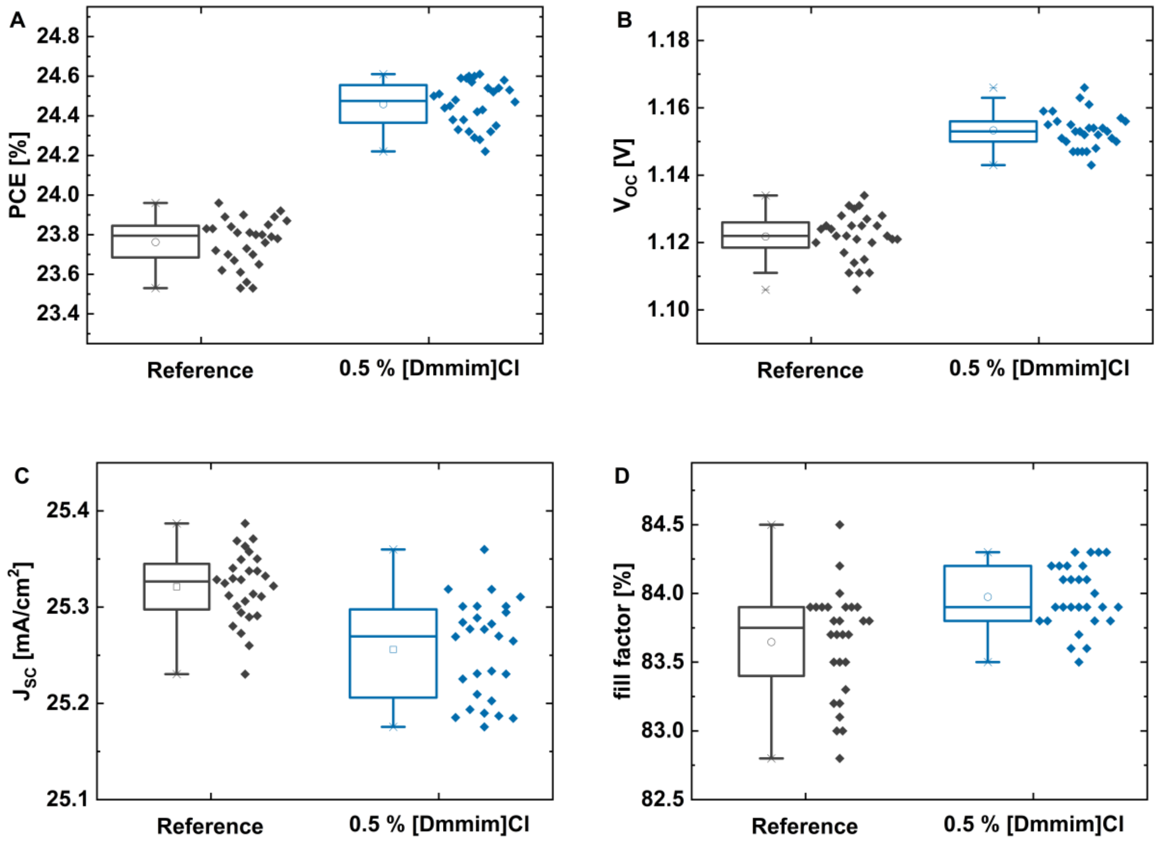

Figure S6: Device parameters of 28 reference and [Dmmim]Cl-doped perovskite solar cells. **(a)** power conversion efficiency (PCE), **(b)** open-circuit voltage, **(c)** short-circuit current, **(d)** fill factor.

The maximum power point tracking of both solar cells, the reference and the [Dmmim]Cl-doped, is shown in Figure S7. Both devices reach fast their equilibrium and show stable power output.

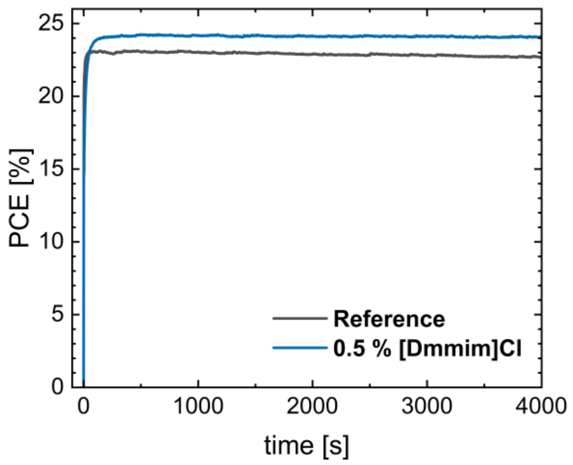

Figure S7: Maximum power point (MPP) tracking of the reference and [Dmmim]Cl-doped solar cell. Both samples, the reference and the 0.5 % [Dmmim]Cl-doped perovskite solar cells, rapidly reach their maximum power output and show a stable power output.

S7 External Quantum Efficiency (EQE) spectra and integrated short-circuit current J_{SC}

The incident photon-to-current conversion (IPCE) values were confirmed as a function of wavelength from 300 to 900 nm (IQE200B, Oriel) for devices without bias light. In order to evaluate the optical properties of the device, no anti-reflection layer was used.

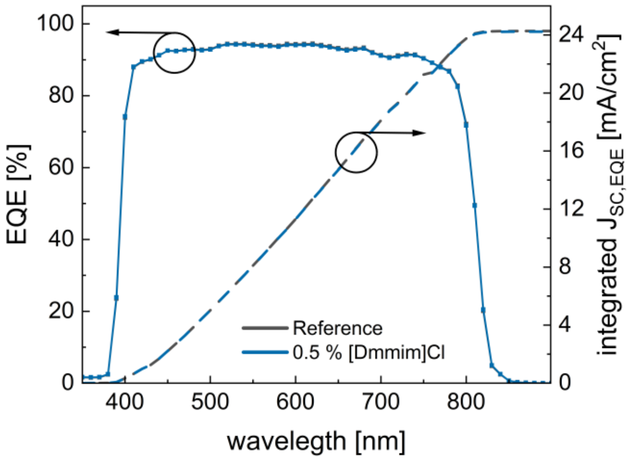

Figure S8: External quantum efficiency (EQE) spectra and the corresponding integrated short-circuit current J_{SC}. Both samples, the reference and the 0.5 % [Dmmim]Cl-doped perovskite solar cells, show only minor differences in their EQE and the resulting integrated J_{SC}. The mismatch between $J_{SC,JV}$ and $J_{SC,EQE}$ is within an acceptable range of 4%.

S8 Temperature-dependent TRMC transients

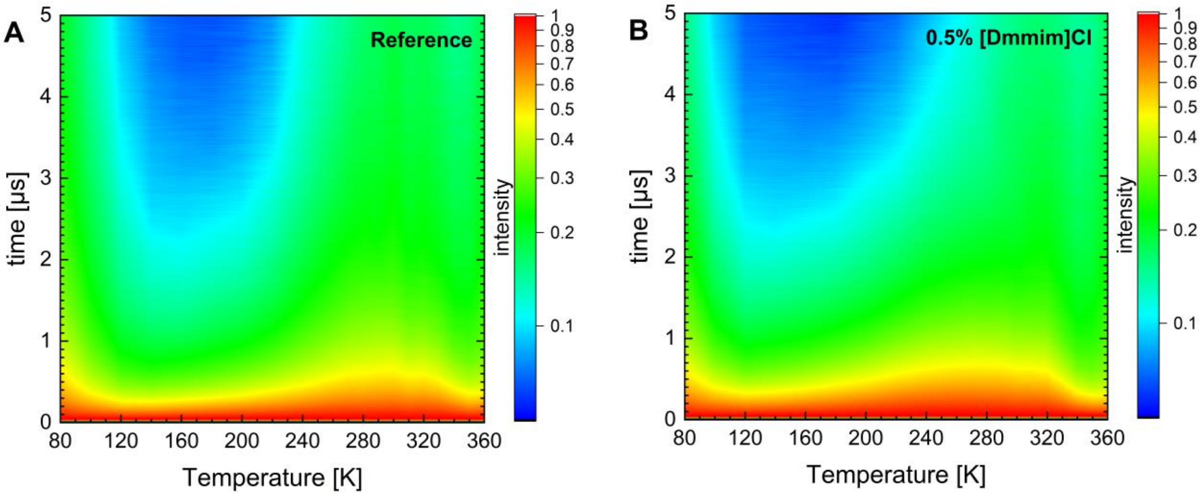

Figure S9: Color plots of TRMC transients measured at different temperatures at the same laser intensity of 3.6E11 photons/cm^2 which corresponds to a charge carrier density of 6.5·10^{15} cm^{-3}. Temperature-dependent behavior of the transients from **(a)** reference and **(b)** [Dmmim]Cl-doped perovskite film. Both show qualitatively the same trend. Starting at 360 K towards lower temperatures, the decay of the transient is decelerated. Between 280 and 240 K, the transient start to decay faster. Around 140 and 120 K, the trend changes again by showing a slower decay, which can be explained by a possible phase transition.

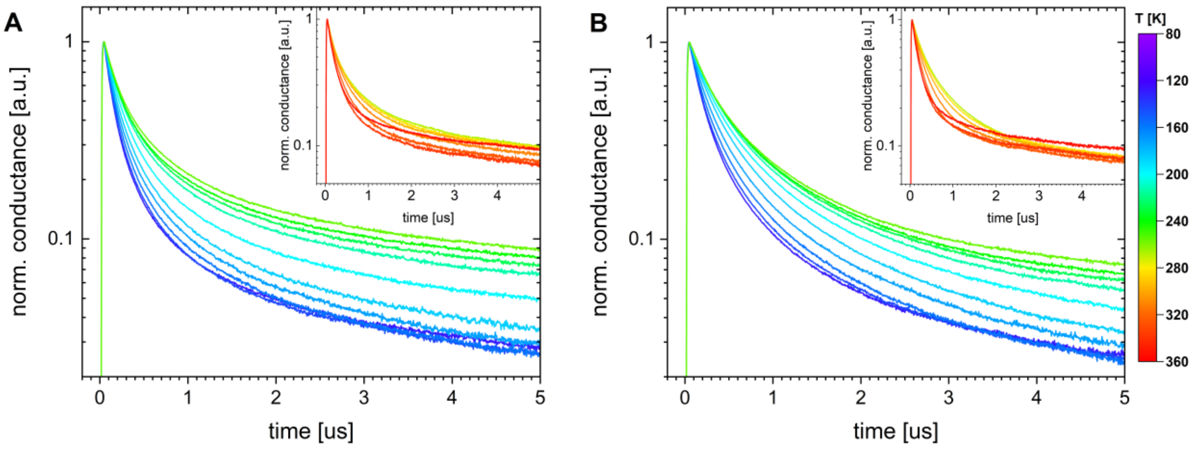

Figure S10: TRMC transients measured at different temperatures (in K) at a laser intensity of 3.6E11 photons/cm^2 which corresponds to a charge carrier density of 6.5·10^{15} cm^{-3}. Temperature-dependent behavior of the transients from **(a)** reference and the

(b) [Dmmim]Cl-doped perovskite film. The inset shows the behavior of the higher temperatures, whereas the transients from green to blue shows the decay of the lower temperatures. The overall trend is the same for the reference and the [Dmmim]Cl-doped perovskite. However, it can be seen, that the initial decay of the doped perovskite is slightly slower compared to the reference.

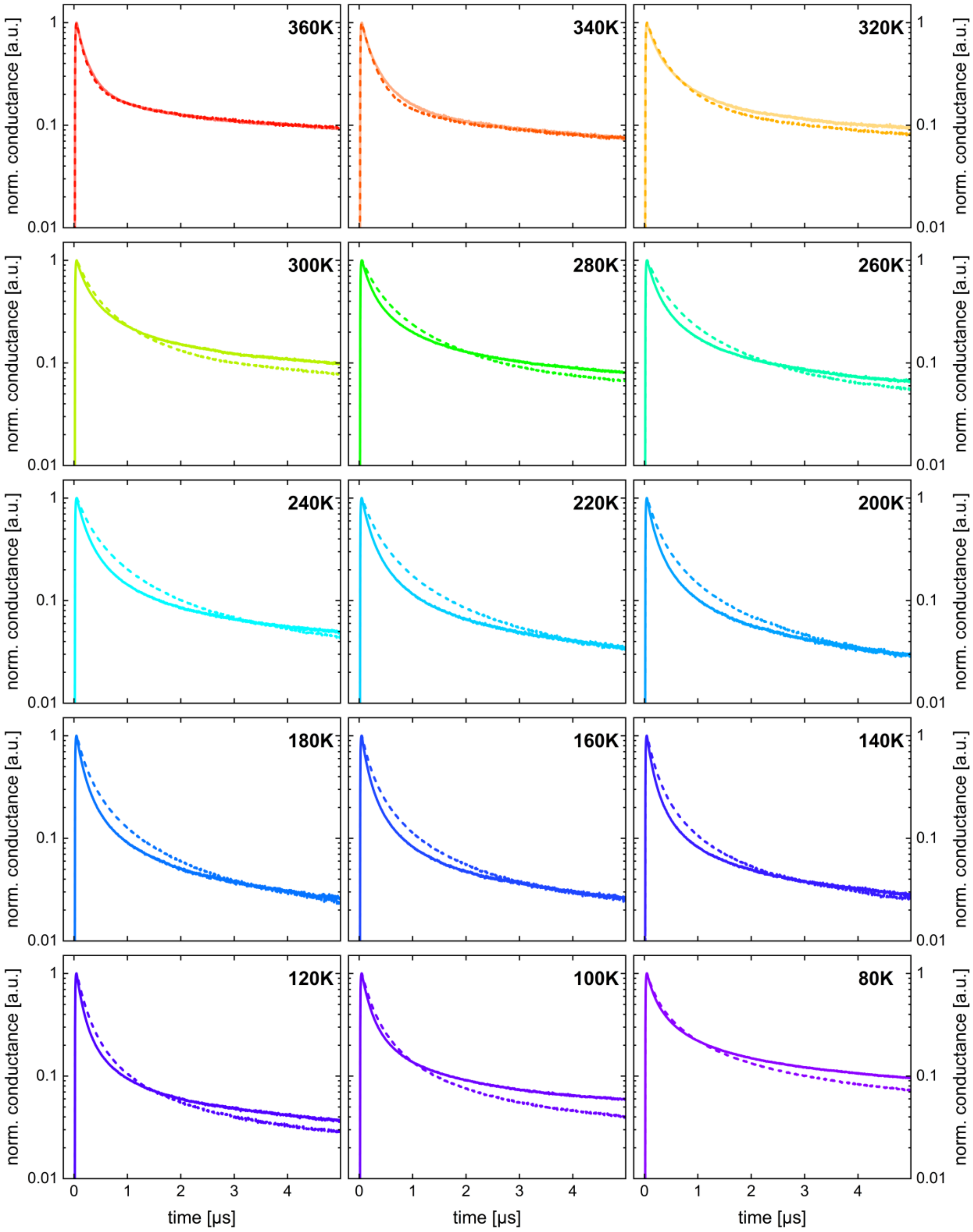

Figure S11: Temperature-dependent transients for reference (line) and [Dmmim]Cl-doped (dashed line) perovskite films (same dataset as in figure S9 and S10). Excited with a laser intensity of 3.6E11 photons/cm^2 which corresponds to a charge carrier density of $6.5\cdot10^{15}$ cm^{-3}.

S9 Photoluminescence quantum yield measurements (PLQY)

For the PLQY measurements, we used an integrating sphere in an Edinburgh Instruments FLS 980. The sample was excited with a 635nm CW laser diode with 98.7 mW/cm^2. The spot size was 0.046 cm^2.

With the PLQY values, we are able to express the loss in quasi-Fermi level splitting (QFLS) induced by the increase of non-radiative recombination in the perovskite layer. [5] As the QFLS represents the maximum achievable open-circuit voltage V_{OC} in a solar cell, conclusions can be drawn about the possible loss mechanism. In the case of medium excitation intensities up to several suns and negligible bending of the quasi-Fermi level between perovskite and electrodes, one can equate the ΔQFLS and the ΔqV_{OC}. [5]

$$\mathrm{QFLS} = \mathrm{QFLS}_{\mathrm{rad}} - k_B T \cdot \ln\left(\frac{1}{\mathrm{PLQY}}\right) \quad \text{(SE7)}$$

$$\Delta\mathrm{QFLS} = k_B T \cdot \ln(\mathrm{PLQY}_1) - k_B T \cdot \ln(\mathrm{PLQY}_2) \quad \text{(SE8)}$$

Supporting Table S1: Absorptance and Photoluminescence Quantum Yield (PLQY) of the reference and [Dmmim]Cl-doped perovskite films after different annealing times at 360 K. Measured at room temperature.

Annealing time (h) at 360 K	Reference perovskite film		[Dmmim]Cl-doped perovskite film	
	Absorptance	PLQY	Absorptance	PLQY
0	81 %	18.0 %	78 %	20.3 %
2	79 %	16.2 %	78 %	20.4 %
4	78 %	15.5 %	79 %	20.4 %
19	79 %	8.3 %	79 %	19.8 %
91	81 %	4.9 %	79 %	18.3 %

S10 Open-circuit voltage decay (OCVD) setup and measurements

OCVD measurements were performed by biasing the solar cell with 1.0 V in forward direction for a duration of 100 s and rapidly switching off the bias using a homemade switch and an Agilent 81150A function generator. Subsequently, the open circuit voltage is monitored over a 1 TΩ input impedance amplifier (Femto Messtechnik GmbH) and with an Agilent Infinium

90254A digital storage oscilloscope. Dark J-V measurements are recorded with a 6430 Sub-Femtoamp Remote Sourcemeter (Keithley).

An exemplary OCVD transient is shown in Figure S12a. The dynamics of electronic and ionic species can be assigned to different timescales by the knowledge of drift-diffusion simulations from Fischer et al. and are highlighted with arrows in Figure S12a. [6] Shown by the blue arrow, in early timescales an initial plateau followed by a constant voltage drop can be related to free carrier kinetics. Fast surface recombination, due to the accumulation of excess charges near the transport layers, is followed by the slower recombination of the remaining charges which need to diffuse through the bulk material resulting in an exponential voltage decay. Afterwards, the OCVD transient shows a slowed down decay (shoulder) at later time scales, as indicated by the red arrow. This shoulder originates from mobile ions diffusing from the electron transport layer to the hole transport layer during the voltage decay caused by the electron-hole recombination. As the redistribution of the mobile ions contributes to the device capacitance, the voltage decay is slowed down resulting in the ionic shoulder. At the end, in the seconds range the voltage decay accelerates due to shunt and leakage currents, as shown by the green arrow. The interpretability of the OCVD transients ends by entering the shunt regime.

In order to determine the mobile ion concentration from OCVD and dark J-V measurements, the device capacity needs to be calculated first. This is defined as the charge variation per voltage dQ/dV, which by taking partial time derivatives is equal to the internal current dQ/dt times the inverse time derivative of the voltage $\mathrm{d}t/\mathrm{d}V$:

$$C\big(V(t)\big) = \frac{\mathrm{d}Q}{\mathrm{d}V} = \frac{\mathrm{d}Q}{\mathrm{d}t}\frac{\mathrm{d}t}{\mathrm{d}V} \tag{SE9}$$

The internal current during the OCVD is well approximated by the internal recombination current $I(V)$ measured by a dark J-V sweep. As the latter yields a positive current reading if charge carriers recombine inside the cell, it needs to be equipped with a negative sign when calculating the device capacity. The inverse time derivative of the voltage is simply the time derivative of the OCVD:

$$C\big(V(t)\big) \approx -I\big(V(t)\big)\left(\frac{\mathrm{d}V_{oc}(t)}{\mathrm{d}t}\right)^{-1} \tag{SE10}$$

Integrating this device capacity over the voltage and normalizing it to the volume of the perovskite layer finally results in the mobile ion concentration:

$$N_{ion} = \frac{1}{A*d}\int C(V)\mathrm{d}V \tag{SE11}$$

as mobile ions are by far the dominant contribution to the device capacity.

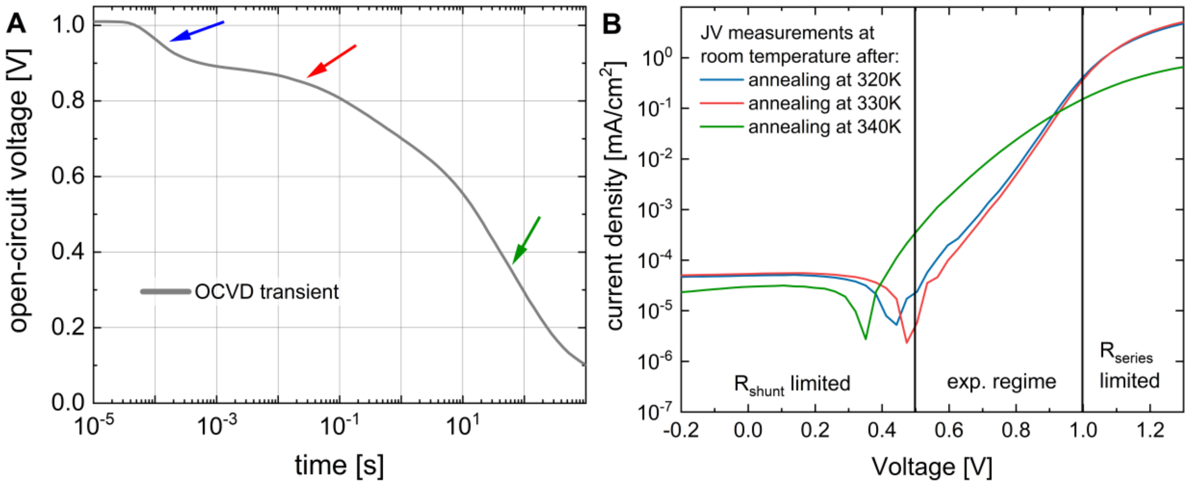

Figure S12: Exemplary open-circuit voltage decay with arrows indicating distinct regions and dark JV after annealing at different temperatures. (a) Blue arrow: Electron-hole recombination starts to occur. **Red arrow:** Ionic shoulder – Redistribution of mobile ions leads to reduced drop in the open-circuit voltage. **Green arrow:** Shunt regime – Leakage currents accelerate decay of the open-circuit voltage. **(b)** After treatment at 320 K and 330 K: Device shows diode-like behavior within the marked regimes. After treatment at 340 K: Series resistance of the cell is greatly increased (drop in current at high voltages). Regime of interest (region of ionic shoulder 0.6 V - 0.9 V) does no longer show an exponential increase in current, thus the dark J-V sweep is no longer a good approximation for the internal recombination current and the mobile ion density can no longer be accurately determined with the used technique.

S11 X-Ray Diffraction (XRD) measurements

The phase composition of perovskite films over time was characterized by XRD with a Bruker D8 Advance diffractometer and a Cu Kα radiation source (λ=1.5418 Å).

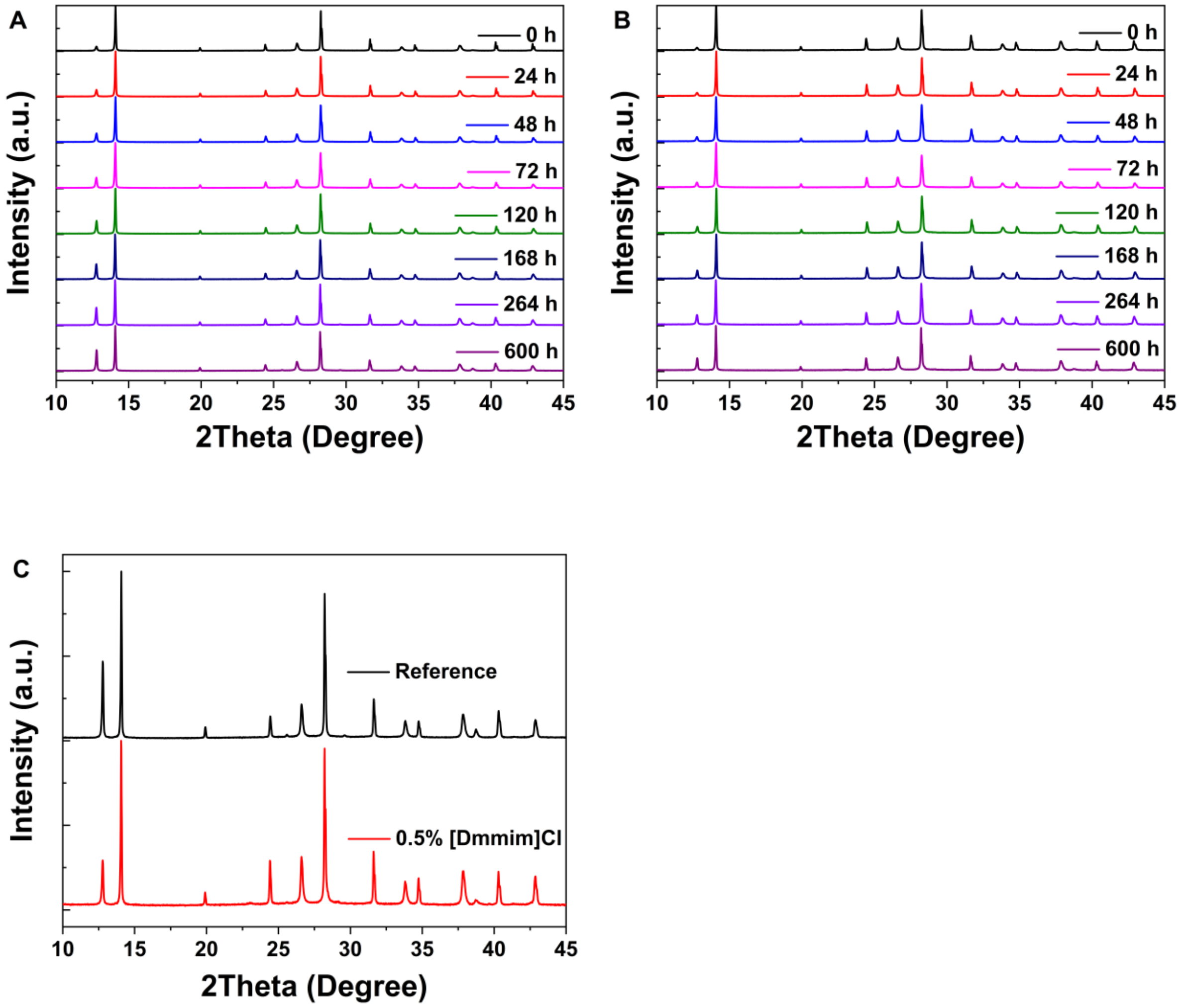

Figure S13: XRD measurements of the reference and [Dmmim]Cl-doped perovskite films, after thermal aging. The reference perovskite film **(a)** shows a pronounced PbI_2 peak between 12.5° and 13° after thermal aging at 330 K, indicating a degradation of the perovskite material. The doped perovskite film **(b)** also shows an increase of the PbI_2 peak, with increasing time. However, the direct comparison of the reference and doped perovskite film after 600 hours of thermal aging as seen in **(c)**, exhibit a lower intensity of the PbI_2 peak. This points towards suppressed decomposition in the [Dmmim]Cl-doped perovskite film.

Supporting Table S2: Photovoltaic performance of the reference devices

Cell	V_{OC} [V]	J_{SC} [mA/cm^2]	FF [%]	PCE [%]
**1**	1.121	25.36	83.5	23.73
**2**	1.125	25.35	83.5	23.81
**3**	1.125	25.34	83.8	23.90
**4**	1.111	25.31	83.8	23.56
**5**	1.114	25.30	83.7	23.61
**6**	1.122	25.23	83.1	23.53
**7**	1.127	25.26	83.2	23.70
**8**	1.131	25.33	83.0	23.80
**9**	1.130	25.34	83.2	23.81
**10**	1.128	25.33	83.3	23.80
**11**	1.134	25.31	83.0	23.84
**12**	1.131	25.29	82.8	23.67
**13**	1.120	25.27	83.5	23.65
**14**	1.122	25.39	83.7	23.85
**15**	1.115	25.31	84.0	23.70
**16**	1.125	25.35	83.7	23.89
**17**	1.124	25.33	83.9	23.89
**18**	1.128	25.31	83.9	23.96
**19**	1.125	25.36	83.9	23.92
**20**	1.122	25.32	83.9	23.83
**21**	1.124	25.29	83.9	23.87
**22**	1.121	25.34	83.7	23.76
**23**	1.111	25.32	83.9	23.62
**24**	1.120	25.33	83.8	23.79
**25**	1.106	25.37	83.9	23.53
**26**	1.121	25.37	83.8	23.83
**27**	1.111	25.29	84.5	23.72
**28**	1.117	25.28	84.2	23.78
**Aver.**	**1.120**	**25.32**	**83.7**	**23.76**

Supporting Table S3: Photovoltaic performance of the 0.5 mol % [Dmmim]Cl-doped devices

Cell	V_{OC} [V]	J_{SC} [mA/cm^2]	FF [%]	PCE [%]
**1**	1.147	25.28	84.2	24.42
**2**	1.152	25.29	84.3	24.57
**3**	1.153	25.32	84.3	24.60
**4**	1.147	25.30	84.2	24.43
**5**	1.154	25.29	84.3	24.59
**6**	1.143	25.28	84.1	24.29
**7**	1.153	25.28	84.2	24.54
**8**	1.154	25.30	84.3	24.60
**9**	1.155	25.30	84.2	24.59
**10**	1.152	25.31	84.1	24.52
**11**	1.150	25.32	84.1	24.48
**12**	1.154	25.36	83.9	24.54

**13**	1.151	25.28	84.1	24.45
**14**	1.147	25.27	83.9	24.32
**15**	1.148	25.19	83.9	24.28
**16**	1.166	25.20	83.7	24.61
**17**	1.153	25.19	83.9	24.38
**18**	1.163	25.19	84.0	24.58
**19**	1.147	25.18	83.8	24.22
**20**	1.156	25.19	83.9	24.44
**21**	1.151	25.18	83.9	24.33
**22**	1.161	25.27	83.6	24.53
**23**	1.159	25.23	83.8	24.51
**24**	1.150	25.23	83.8	24.32
**25**	1.155	25.26	83.6	24.38
**26**	1.157	25.23	83.8	24.47
**27**	1.159	25.21	83.9	24.50
**28**	1.156	25.23	83.5	24.35
**Aver.**	**1.150**	**25.26**	**84.0**	**24.46**

References

[1] Y. Ding, B. Ding, H. Kanda, O.J. Usiobo, T. Gallet, Z. Yang, Y. Liu, H. Huang, J. Sheng, C. Liu, Y. Yang, V.I.E. Queloz, X. Zhang, J.-N. Audinot, A. Redinger, W. Dang, E. Mosconic, W. Luo, F. de Angelis, M. Wang, P. Dörflinger, M. Armer, V. Schmid, R. Wang, K.G. Brooks, J. Wu, V. Dyakonov, G. Yang, S. Dai, P.J. Dyson, and M.K. Nazeeruddin, *Nature nanotechnology*, **2022**, Vol. 17, 598–605.

[2] L.-L. Gao, L.-S. Liang, X.-X. Song, B. Ding, G.-J. Yang, B. Fan, C.-X. Li, and C.-J. Li, *J. Mater. Chem. A*, **2016**, Vol. 4, 3704–3710.

[3] M.B. Johnston, and L.M. Herz, *Accounts of chemical research*, Vol. 49, 146–154, **2016**.

[4] F. Ruf, M.F. Aygüler, N. Giesbrecht, B. Rendenbach, A. Magin, P. Docampo, H. Kalt, and M. Hetterich, *APL Materials*, **2019**, Vol. 7, 31113.

[5] P. Caprioglio, M. Stolterfoht, C.M. Wolff, T. Unold, B. Rech, S. Albrecht, and D. Neher, *Adv. Energy Mater.*, **2019**, Vol. 9, 1901631.

[6] M. Fischer, D. Kiermasch, L. Gil-Escrig, H.J. Bolink, V. Dyakonov, and K. Tvingstedt, *Sustainable Energy Fuels*, **2021**, Vol. 5, 3578–3587.
